# Supplementary material for: miR-205 Regulates the Fusion of Porcine Myoblast by Targeting the Myomaker Gene
Source: Cells. 2023 Apr 7;12(8):1107. doi: 10.3390/cells12081107 (PMC10136817; doi:10.3390/cells12081107)
Supplement: Supplementary file 1 [file cells-12-01107-s001.zip › Table S1.pdf]

Table S1. MiRNAs studied and correlation analysis of miRNAs and Myomaker gene

miR-name	primer (5' to 3')	correlation (*r*)
ssc-miR-491	AGTGGGGAACCCTTCCATGAGG	-0.084
ssc-miR-205	TCCTTCATTCCACCGGAGTCTG	-0.538
ssc-miR-30b-3p	CTGGGAGGTGGATGTTTACTT	-0.181
ssc-miR-30c-3p	CTGGGAGAAGGCTGTTTACTCT	-0.007
ssc-mir-92b-5p	AGGGACGGGACGCGGTGCAGTGTT	0.066
ssc-miR-365-5p	GAGGGACTTTCAGGGGCAGCTGT	0.065
ssc-miR-542-5p	TCGGGGATCATGTCACGA	0.175
ssc-miR-582-3p	TAACCGGTTGAACAACTGAACC	0.111
