# Supplementary material for: miR-205 Regulates the Fusion of Porcine Myoblast by Targeting the Myomaker Gene
Source: Cells. 2023 Apr 7;12(8):1107. doi: 10.3390/cells12081107 (PMC10136817; doi:10.3390/cells12081107)
Supplement: Supplementary file 1 [file cells-12-01107-s001.zip › Table S2.pdf]

Table S2. Prediction of binding sites of miRNAs and 3' UTR of Myomaker

miR-name	binding sites (3' to 5')	minimal free energy (kcal/mol)
ssc-miR-491	GGGGUG	-27.6
ssc-miR-205	ACUUCCU	-27.7
ssc-miR-30b-3p	GGGU	-26.5
ssc-miR-30c-3p	GAGGGUC	-23.5
