# Supplementary material for: miR-205 Regulates the Fusion of Porcine Myoblast by Targeting the Myomaker Gene
Source: Cells. 2023 Apr 7;12(8):1107. doi: 10.3390/cells12081107 (PMC10136817; doi:10.3390/cells12081107)
Supplement: Supplementary file 1 [file cells-12-01107-s001.zip › Table S3.pdf]

Table S3. Validation reporting for miR-205-Myomaker interaction

1	Gene	Gene name	Myomaker (Tmem8c)
		Entrez ID	100627299
2	Target miRNA	Gene name	miR-205
		Entrez ID	100316607
3	Species	Species	Sus scrofa
		Species ID	9823
4	Experimental validation of miRNA–target interaction	Sequence of the target region, 5'-3'	TGAAGG
		Genomic location of 3'UTR	213-218
		Method for experimental validation	luciferase reporter assay, qPCR
		Tissue, cells	longissimus dorsi, primary MSCs
5	Sequence variant	rs number (synonym)	na
6	Associated disease or phenotype	As named in the reference	muscular dystrophy, sarcopenia,
		DOID (if available)	na
7	Reference	Author, year	na
		PMID	na
