# Supplementary material for: miR-205 Regulates the Fusion of Porcine Myoblast by Targeting the Myomaker Gene
Source: Cells. 2023 Apr 7;12(8):1107. doi: 10.3390/cells12081107 (PMC10136817; doi:10.3390/cells12081107)
Supplement: Supplementary file 1 [file cells-12-01107-s001.zip › Table S4.pdf]

Table S4. Gene-specific primers used for qRT-PCR and 3' RACE

Gene name	Sequence (5' to 3')	TM/℃
Myomaker	F-TGATGTTTGGCGTCCTGACC<br>R-AAGAAAAAGCGCAGCATGAGG	61.4
MyoG	F-AGGCTACGAGCGGACTGAG<br>R-GCAGGGTGCTCCTCTTCAG	62.5
MyoD	F-AAGTCAACGAGGCCTTCGAG<br>R-GGGGGCCGCTATAATCCATC	53.7
Myf5	F-GATCAGCAACTCCGAGCAAC<br>R-ATGAGCCTGGAACTAGACGC	53.7
Pax7	F-CAGTGAGTTCGATTAGCCGC<br>R-AGATCGGGTTCTGACTCCAC	58
MyH1	F-GCACCGTGGACTACAACATC<br>R-TGAGCAGCTTGAGAGAGGAC	56.5
MyH2a	F-GGAGATCGACGACCTTGCTA<br>R-CTCCTTGGATTTCAGCTCGC	
MyH2b	F-CTGAAGGACACTCAGCTCCA<br>R-GCTCTTCAATCTCAGCCTGC	
MyH2x	F-TGAGGAAGCGGAGGAACAAT<br>R-TGAACCTCCCGACTCTTGAC	
GAPDH	F- CCCCTTCATTGACCTCCACT<br>R- CCATTTGATGTTGGCGGGAT	59.4
β-Actin	F-TCTGGCACCACACCTTCT<br>R-TGATCTGGGTCATCTTCTCAC	
3′ RACE Oligo(dT)-anchor primer	GCTGTCAACGATACGCTACGTAAC<br>GGCATGACAGTGTTTTTTTTTTTTT	66,68,70
3′ Myomaker specific primer	TACACACAGCAGATAGGCCC	
3' adaptor outer primer	TACCGTCGTTCCACTAGTGATTT	
ssc-miR-491	AGTGGGGAACCCTTCCATGAGG	60
ssc-miR-205	TCCTTCATTCCACCGGAGTCTG	
ssc-miR-30b-3p	CTGGGAGGTGGATGTTTACTT	
ssc-miR-30c-3p	CTGGGAGAAGGCTGTTTACTCT	
ssc-mir-92b-5p	AGGGACGGGACGCGGTGCAGTGTT	
ssc-miR-365-5p	GAGGGACTTTCAGGGGCAGCTGT	
ssc-miR-542-5p	TCGGGGATCATGTCACGA	
ssc-miR-582-3p	TAACCGGTTGAACAACTGAACC	
Uni-miR qPCR primer	included in kit	
U6	F-CTCGCTTCGGCAGCACA<br>R-AACGCTTCACGAATTTGCGT	
