# Supplementary figures and images for: miR-205 Regulates the Fusion of Porcine Myoblast by Targeting the Myomaker Gene
Source: Cells. 2023 Apr 7;12(8):1107. doi: 10.3390/cells12081107 (PMC10136817; doi:10.3390/cells12081107)

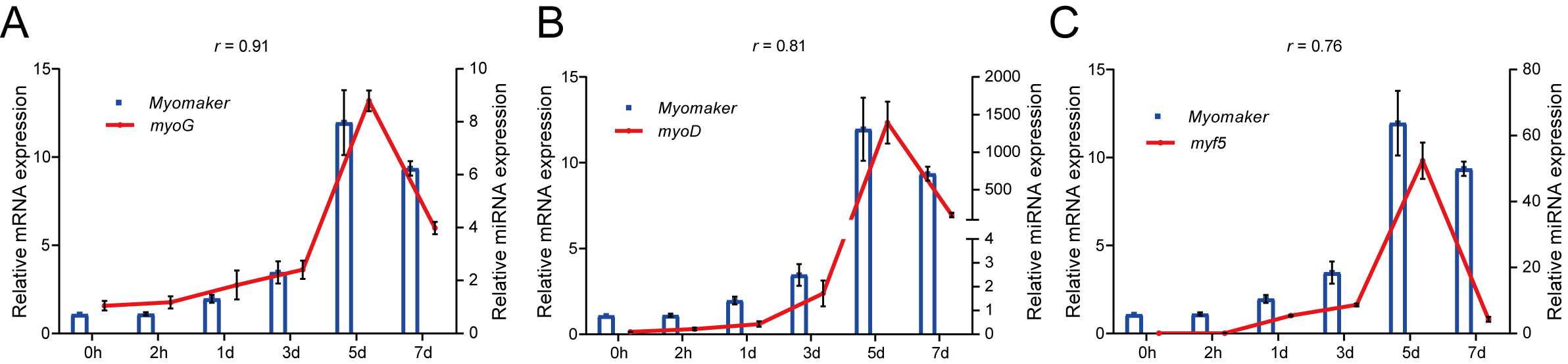

Supplement: Supplementary file 1 [file cells-12-01107-s001.zip › Figure S1.tif]
